# Author Correction: Effects of drought and recovery on soil volatile organic compound fluxes in an experimental rainforest

**DOI:** 10.1038/s41467-023-42207-4

**Published:** 2023-10-11

**Authors:** Giovanni Pugliese, Johannes Ingrisch, Laura K. Meredith, Eva Y. Pfannerstill, Thomas Klüpfel, Kathiravan Meeran, Joseph Byron, Gemma Purser, Juliana Gil-Loaiza, Joost van Haren, Katerina Dontsova, Jürgen Kreuzwieser, S. Nemiah Ladd, Christiane Werner, Jonathan Williams

**Affiliations:** 1 Ecosystem Physiology, Faculty of Environment and Natural Resources, University of Freiburg, Freiburg, Germany; 2 Atmospheric Chemistry Department, Max Planck Institute for Chemistry, Mainz, Germany; 3 Universität Innsbruck, Department of Ecology, Innsbruck, Austria; 4 School of Natural Resources and the Environment, University of Arizona, Tucson, AZ USA; 5 Biosphere 2, University of Arizona, Oracle, AZ USA; 6 UK Centre for Ecology & Hydrology, Penicuik, Edinburgh, UK; 7 School of Chemistry, The University of Edinburgh, Edinburgh, UK; 8 Department of Environmental Science, University of Arizona, Tucson, AZ USA; 9 Department of Environmental Sciences, University of Basel, Basel, Switzerland; 10 Climate and Atmosphere Research Center, The Cyprus Institute, Nicosia, Cyprus; 11 Present Address: Department of Environmental Science, Policy, and Management, University of California at Berkeley, Berkeley, CA USA

**Keywords:** Carbon cycle, Atmospheric chemistry

Correction to: *Nature Communications* 10.1038/s41467-023-40661-8, published online 21 August 2023

The original version of the supplementary information associated with this article contained an error in the title, which incorrectly read ‘Effects of drought and recovery on volatile organic compound soil fluxes in an experimental rainforest’. The correct version replaces this title with ‘Effects of drought and recovery on soil volatile organic compound fluxes in an experimental rainforest’. The HTML has been updated to include a corrected version of the supplementary information.

The original version of this Article contained an error in reference 70, which incorrectly read ‘Effects of drought and recovery on volatile organic compound soil fluxes in an experimental rainforest’. The correct version replaces this with ‘Effects of drought and recovery on soil volatile organic compound fluxes in an experimental rainforest’. This has been corrected in both the PDF and HTML versions of the Article.

### Supplementary information

Updated Supplementary Information

