# Supplementary material for: Effects of drought and recovery on soil volatile organic compound fluxes in an experimental rainforest
Source: Nat Commun. 2023 Aug 21;14:5064. doi: 10.1038/s41467-023-40661-8 (PMC10442410; doi:10.1038/s41467-023-40661-8)
Supplement: Supplementary file 1 — Supplementary Information [file 41467_2023_40661_MOESM1_ESM.pdf]

Supplementary Information for

Effects of drought and recovery on soil volatile organic compound fluxes in an experimental rainforest

Giovanni Pugliese[1,2]*, Johannes Ingrisch[1,3], Laura K. Meredith[4,5], Eva Y. Pfannerstill[2#], Thomas Klüpfel[2], Kathiravan Meeran[3], Joseph Byron[2], Gemma Purser[6, 7], Juliana Gil-Loaiza[4], Joost van Haren[4,5], Katerina Dontsova[5,8], Jürgen Kreuzwieser[1], S. Nemiah Ladd[1,9], Christiane Werner[1], and Jonathan Williams[2,10]

[1]Ecosystem Physiology, Faculty of Environment and Natural Resources, University of Freiburg, Freiburg, Germany

[2]Atmospheric Chemistry Department, Max Planck Institute for Chemistry, Mainz, Germany

[3]Universität Innsbruck, Department of Ecology, Innsbruck, Austria,

[4]School of Natural Resources and the Environment, University of Arizona, Tucson, Arizona, USA

[5]Biosphere 2, University of Arizona, Oracle, AZ, USA

[6]UK Centre for Ecology & Hydrology, Penicuik, Edinburgh, UK

[7]School of Chemistry, The University of Edinburgh, Edinburgh, UK

[8]Department of Environmental Science, University of Arizona, Tucson, Arizona, USA

[9]Department of Environmental Sciences, University of Basel, Basel, Switzerland

[10]Climate and Atmosphere Research Center, The Cyprus Institute, Nicosia, Cyprus

[#]now at: Department of Environmental Science, Policy, and Management, University of California at Berkeley, Berkeley, CA, USA

*Corresponding author: g.pugliese@mpic.de (ORCID: https://orcid.org/0000-0002-2869-3588)

The PDF file includes:

Speciation of monoterpenes soil flux

Tables S1 to S4

Figures S1 to S6

Speciation of monoterpenes soil flux

For the speciation of monoterpenes soil fluxes, periodically isolated samples were collected from 3 manual PVC chambers placed at 3 fixed locations of the B2-TRF. Manual PVC chambers consisted of a PVC collar inserted ~ 1cm into the soil surface and a PVC lid containing two Teflon sample valves, an inlet and outlet. The lid was closed for approximately 15 minutes prior to sampling. Samples were collected onto sorbent cartridges from the soil chamber by drawing air (200 ml min^{-1} for ~ 10 minutes) from the outlet through a sorbent cartridge using a handheld pump (SKC ltd., Dorset, U.K). In addition, an ambient air sample was also taken from outside but close to air inlet of the manual chamber. Samples were collected onto inert coated stainless steel cartridges (SilcoNert 2000 (SilcoTek™, Germany)) contained 150 mg of Tenax® TA (Buchem BV, Apeldoorn, The Netherlands) followed by 150 mg of Carbograph™ 5 TD (560 m^2/g) (L.A.R.A s.r.l, Rome, Italy) sorbent. The size of the Carbograph™ particles was in the range of 20-40 mesh. Sample desorption was performed using a two-stage automated thermal desorber (TD100-xr, MARKES International, U.K.), with helium 6.0 as the carrier gas. The sample was desorbed at a temperature of 250 °C and a flow of 50 ml min^{-1} of helium for 10 min, and was pre-concentrated onto a cold trap (materials emissions, MARKES International, U.K.) at -30 °C. The cold trap was purged with carrier gas for 1 min with a flow of 50 ml min^{-1} then rapidly heated to 300 °C. The sample was removed from the cold trap with a flow of 3 ml min^{-1} and injected into the column. The separation of the sampled compounds was achieved using a 30 m β-DEX™ 120 column (Sigma-Aldrich Chemie GmbH, Germany) with 0.25 mm internal diameter and a film thickness of 0.25 µm. The temperature program used was as follows, 40 °C for 5 minutes then 40 °C to 150 °C at 4 °C min^{-1} and 150 °C to 200 °C at 30 °C min^{-1}. The column flow was set to 1 ml min^{-1}. Detection was achieved using a time of flight mass spectrometer (Bench TOF-Select, MARKES International, U.K.). A standard gas calibration mixture (Apel-Riemer 2019) containing all measured monoterpenes was used to identify and calibrate the samples.

The soil fluxes were calculated as:

$$F\ [\mu mol\ m^{-2}\ s^{-1}] = \frac{P\ f}{R\ T\ S}\ (C_{out} - C_{in}) \qquad (1)$$

Where P is the pressure inside the chamber (Pa), f is sampling air flow throughout the chamber (m^3 s^{-1}), R is the gas constant (8.314 m^3 Pa K^{-1} mol^{-1}), T is the chamber air temperature (K), S is the soil surface area within the chamber (m^2), C_{out} is the compound concentration in the ambient air (ppm), and C_{in} is the compound concentration inside the soil chamber (ppm).

Table S 1 Volatile organic compound (VOC) gas standard for daily calibration included in the two calibration standard cylinders with their respective detection limit (LOD) and total uncertainty. LOD was determined as the 3σ uncertainty of the zero calibration.

Compound	Protonated ion	$(m/z)_{exact}$	Concentration Cylinder 1 [ppbV]	Concentration Cylinder 2 [ppbV]	LOD range [pptV]	Total uncertainty [%]
Methanol	CH_5O^+	33.0335	494	433	145 - 524	9
Acetonitrile	$C_2H_4N^+$	42.0338	487	461	56 - 209	9
Ethanol	$C_2H_7O^+$	47.0491	504	0	319 - 815	11
Propanal	$C_3H_7O^+$	59.0491	0	463	123 - 364	6
Acetone	$C_3H_7O^+$	59.0491	497	0	106 - 331	6
DMS	$C_2H_7S^+$	63.0263	537	0	53 - 289	7
Isoprene	$C_5H_9^+$	69.0699	509	0	56 - 233	6
Methacrolein, methyl vinyl ketone	$C_4H_7O^+$	71.0491	252, 243	0	56 - 199	6
Benzene	$C_6H_7^+$	79.0542	510	497	28 - 98	6
Toluene	$C_7H_9^+$	93.0699	489	0	31 - 195	6
Furfural	$C_5H_5O_2^+$	97.0284	479	0	78 - 791	9
Hexanal	$C_6H_{13}O^+$	101.0961	0	494	58 - 160	9
Styrene	$C_8H_9^+$	105.0699	0	498	44 - 110	6
m-Xylene	$C_8H_{11}^+$	107.0855	0	499	37 - 87	6
Acetophenone	$C_8H_9O^+$	121.0648	0	496	117 - 576	11
p-Isopropyltoluene	$C_{10}H_{15}^+$	47.0491	0	474	140 - 280	8
α-Pinene, β-Pinene, 3-Carene, Limonene	$C_{10}H_{17}^+$	137.1325	0	126, 125, 143, 148	58 - 122	6

Linalool	$C_{10}H_{19}O^+$	155.1430	466	0	253 - 925	16
α-Cedrene	$C_{15}H_{25}^+$	205.1951	105	0	21 -94	12
Hexamethylcyclotrisiloxane (D3)	$C_6H_{19}O_3Si_4^+$	251.0406	502	509	15 - 54	6
Octamethylcyclotetrasiloxane (D4)	$C_8H_{25}O_4Si_4^+$	297.0824	502	499	14 - 74	12
Decamethylcyclopentasiloxane (D5)	$C_{10}H_{31}O_5Si_5^+$	371.1012	518	498	18 - 336	23
Dodecamethylcyclohexasiloxane (D6)	$C_{12}H_{37}O_6Si_6^+$	445.1200	190	189	56 - 1530	13

Table S 2 List of tentatively identified volatile organic compounds (VOCs) that showed discernable soil fluxes.

$(m/z)_{measured}$	$(m/z)_{exact}$	Mass accuracy [ppm]	Protonated ion	Potential compound
33.0331	33.0335	-12.11	CH_5O^+	Methanol
45.0332	45.0335	-6.66	$C_2H_4O^+$	Acetaldehyde
47.0488	47.0491	-6.38	$C_2H_7O^+$	Ethanol
49.0126	49.0106	40.80	CH_5S^+	Methanethiol
59.0498	59.0491	11.85	$C_3H_7O^+$	Acetone/propanal
62.029*	62.0236	87.06	CH_4ONO^+	Methyl nitrite
63.0255	63.0263	-12.69	$C_2H_7S^+$	Dimethyl sulfide
69.07	69.0699	1.45	$C_5H_9^+$	Isoprene
71.0493	71.0491	2.81	$C_4H_7O^+$	Methacrolein/methyl vinyl ketone
73.0649	73.0648	1.37	$C_4H_9O^+$	Butanone/butanal
85.0657	85.0648	10.58	$C_5H_9O^+$	Ethyl vinyl ketone/cyclopentanone/pentenal
87.0809	87.0804	5.74	$C_5H_{11}O^+$	Pentanone/pentanal
91.0555	91.0576	-23.06	$C_4H_{11}S^+$	Isopropyl methyl sulfide
93.0368	93.0369	-1.07	$C_3H_9OS^+$	2-methylthioethanol
137.1320	137.1325	-3.65	$C_{10}H_{17}^+$	Monoterpenes

*subtraction of the contribution from the ^{13}C isotopologues.

Table S 3 Blank fluxes for all investigated volatile organic compounds (VOCs). Mean flux and SD represent the average and the standard deviation, respectively, over three replicate measurements (n =3).

VOC	Mean (n=3) blank flux [µmol m^{-2} h^{-1}]	SD [µmol m^{-2} h^{-1}]
Isoprene	-0.20211	0.24616
C_5H_8O	0.00928	0.00442
Monoterpenes	0.00880	0.01153
Methacrolein	0.00272	0.00090
Acetone fluxes	0.05415	0.00484
Acetaldehyde	0.01805	0.00394
Butanone	0.00923	0.00714
Pentanone	0.01138	0.00326
Methanethiol	0.00990	0.00360
Dimethyl sulfide	-0.00100	0.01353
$C_4H_{10}S$	-0.00009	0.00567
C_3H_8OS	0.02643	0.00581
Methanol	-0.10493	0.03807
Ethanol	0.11547	0.10256
Methyl nitrite	0.00095	0.00422

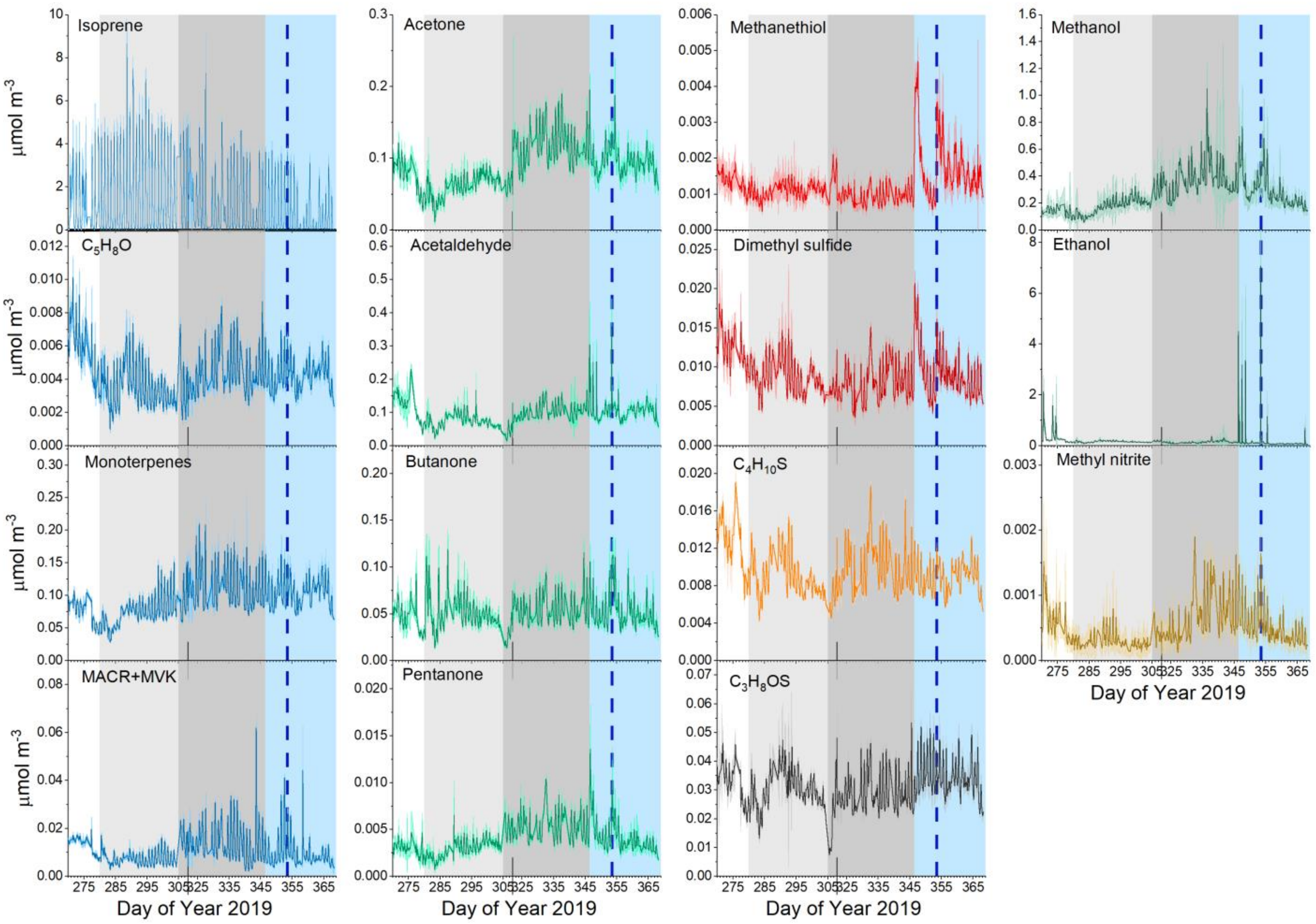

Figure S 1 Time series of ambient volatile organic compound (VOC) concentrations. Ambient VOC concentrations were measured from the soil chambers with open lids during the last minute of the pre-purge. Lines represent averaged concentrations measured over the 12 soil chambers (n = 12) and the shaded areas indicate the standard deviation. Background colors indicate the different phases of the campaign: pre-drought (white, Day of Year 270-279), early drought (light gray, Day of Year 280-305), severe drought (dark gray, Day of Year 305-346), and recovery (light blue, Day of Year 346-369). The first drought-ending rain event occurred at the start of the recovery period, and the vertical blue line indicates the time of the second rain event (Day of Year 353).

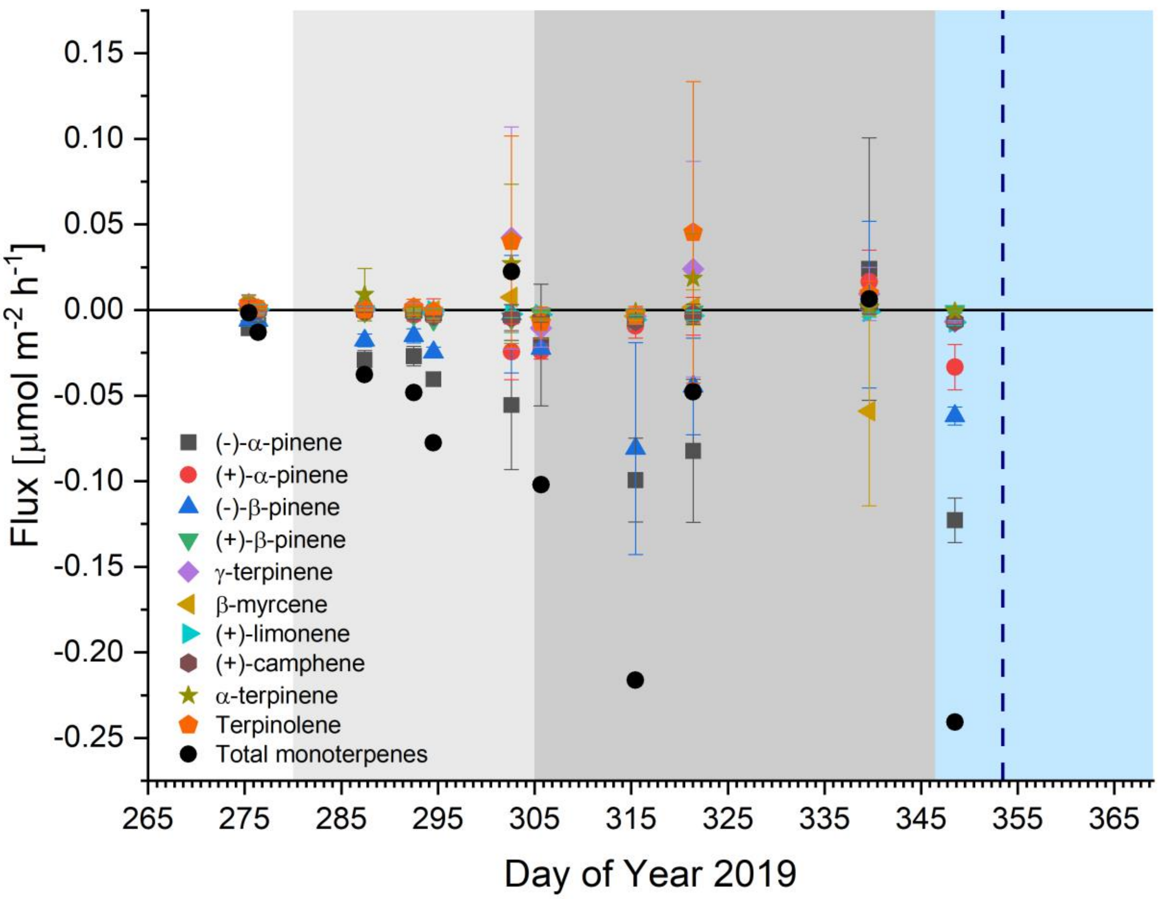

Figure S 2 Soil fluxes of speciated monoterpenes measured by of gas chromatography time of flight mass spectrometry (GC-ToF-MS). Symbols represent averaged fluxes over the 3 manual chambers (n = 3) and error bars indicate the standard deviation. Black circle represents the sum of all monoterpenes flux. Background colors indicate the different phases of the campaign: pre-drought (white, Day of Year 270-279), early drought (light gray, Day of Year 280-305), severe drought (dark gray, Day of Year 305-346), and recovery (light blue, Day of Year 346-369). The first drought-ending rain event occurred at the start of the recovery period, and the vertical blue line indicates the time of the second rain event (Day of Year 353).

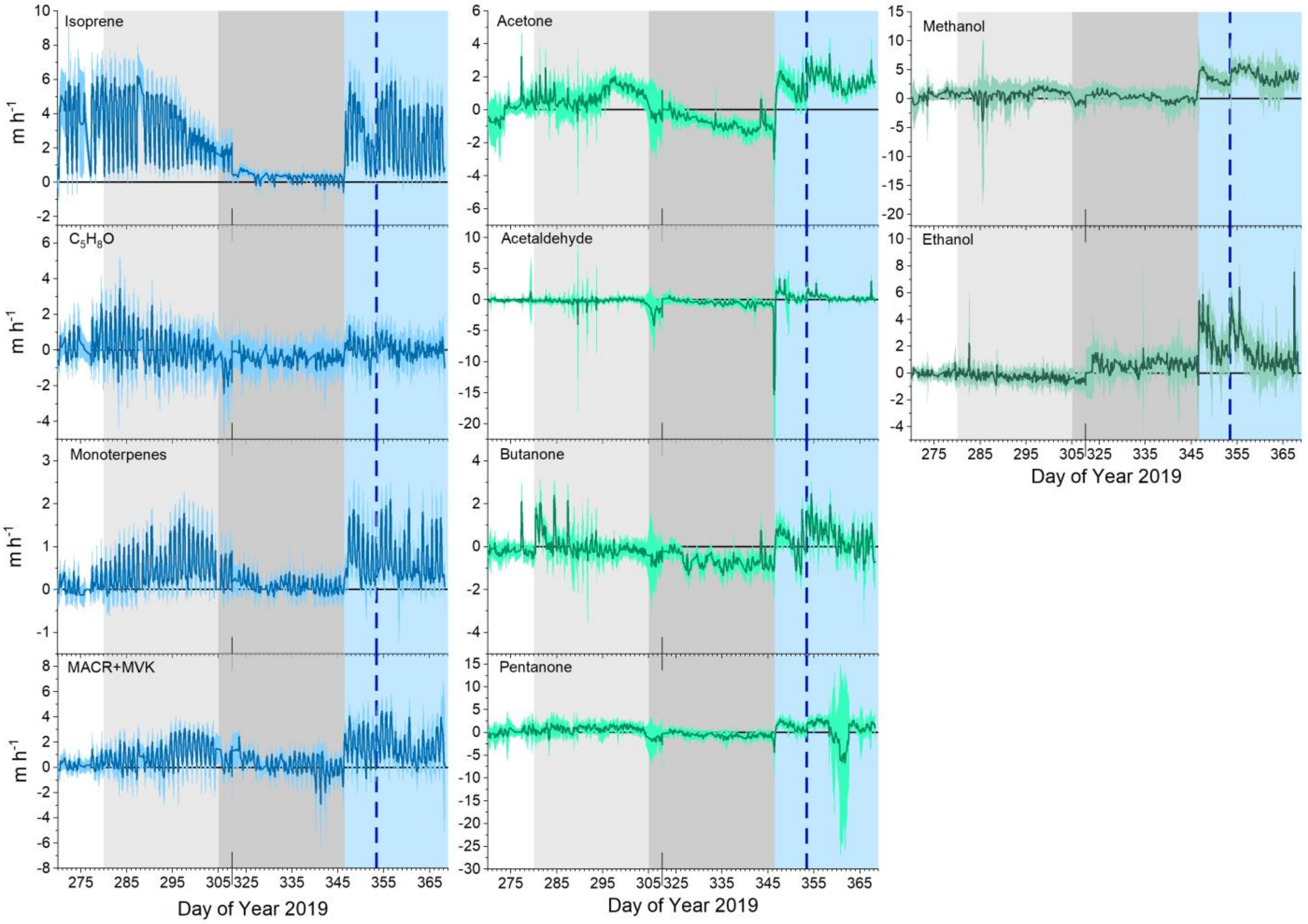

Figure S 3 Time series of deposition velocities for volatile organic compounds (VOCs) that were taken-up by the soil. Lines represent averaged deposition velocities over the 12 soil chambers (n = 12) and the shaded areas indicate the standard deviation. Background colors indicate the different phases of the campaign: pre-drought (white, Day of Year 270-279), early drought (light gray, Day of Year 280-305), severe drought (dark gray, Day of Year 305-346), and recovery (light blue, Day of Year 346-369). The first drought-ending rain event occurred at the start of the recovery period, and the vertical blue line indicates the time of the second rain event (Day of Year 353). Positive deposition velocities represent the concentration-independent VOC uptake capacity of soil, whereas negative deposition velocities are derived from emission fluxes and are not physically meaningful.

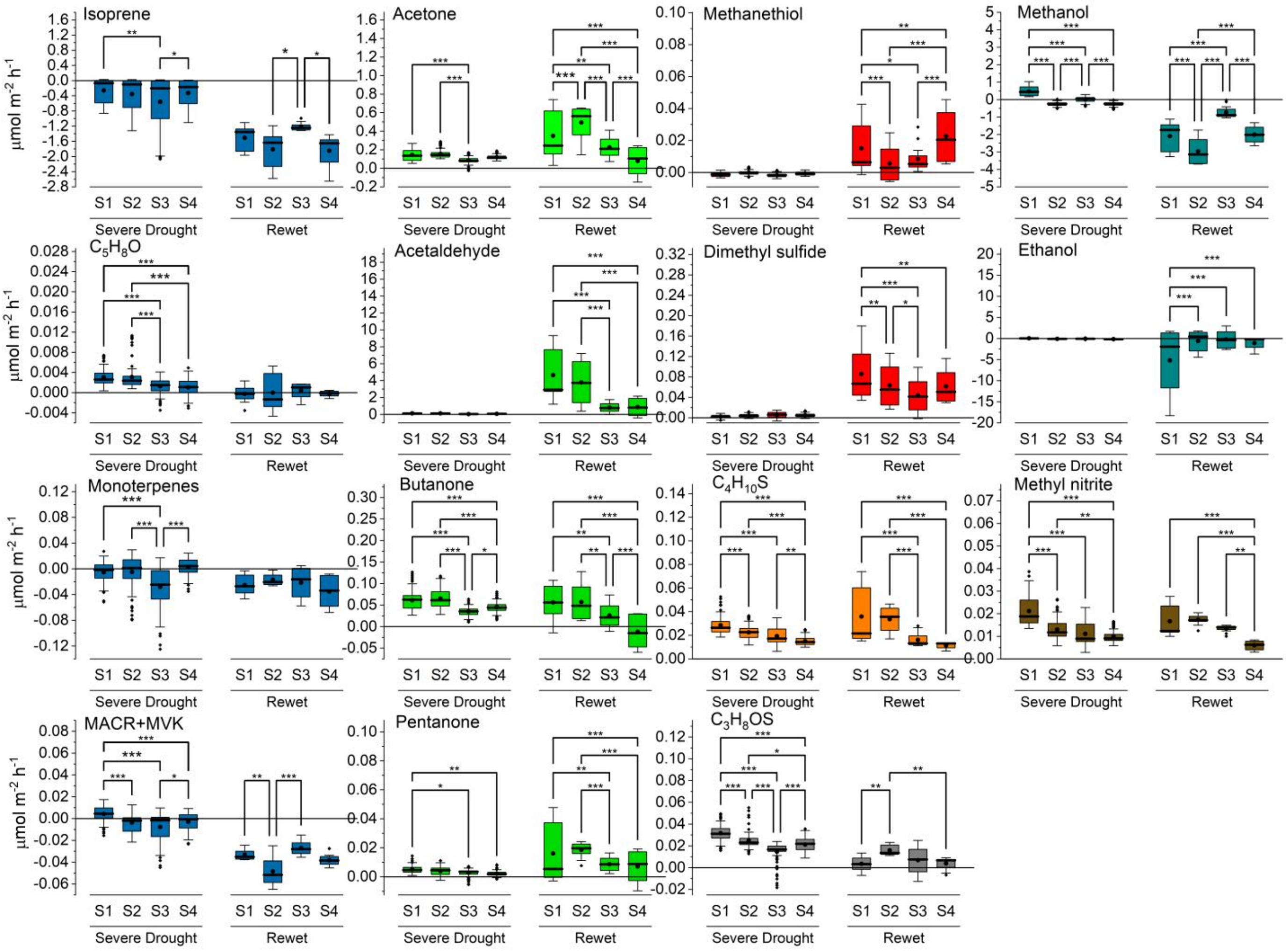

Figure S 4 Soil volatile organic compound (VOC) fluxes from the four different sites of the Biosphere 2 Tropical Rainforest, over the last two days of severe drought and over the first seven hours after the first rain rewet. For severe drought period, VOC fluxes from all 3 chambers placed at each of the 4 sites were considered (n = 81 data points per site). For rewet period, only the 9 chambers (2 chambers for site 1, site 2, and site 3, 3 chambers for site 4) subjected to the rain rewet were considered (n = 12 data points per site). The boxes represent 25% to 75% of the dataset with the filled circle and central lines indicating the mean and median values, respectively. The whiskers indicate the minimum and maximum data points at 1.5 times the interquartile range. Filled diamond indicate the outliers. Statistical differences between soil fluxes from each site were obtained from Tukey mean comparison test which accounts for Family-wise error rate. Significance level was set to p-value <= 0.05 and statistically significant differences are labeled with asterisks as follow: * p-value <=0.05; ** p-value <=0.01; *** p-value <=0.001. The exact p-values from Tukey mean comparison test are reported in Table S4.

Table S 4 Exact p-values from Tukey mean comparison test between soil fluxes from each site of the Biosphere 2 Tropical Rainforest over the last two days of severe drought and over the first seven hours after the first rain rewet. For severe drought period, VOC fluxes from all 3 chambers placed at each of the 4 sites were considered (n = 81 data points per site). For rewet period, only the 9 chambers (2 chambers for site 1, site 2, and site 3, 3 chambers for site 4) subjected to the rain rewet were considered (n = 12 data points per site). Significance level was set to p-value <= 0.05 and statistically significant differences are labeled with asterisks as follow: * p-value <=0.05; ** p-value <=0.01; *** p-value <=0.001.

Compound	Severe drought						Rewet					
	S1 vs S2	S1 vs S3	S1 vs S4	S2 vs S3	S2 vs S4	S3 vs S4	S1 vs S2	S1 vs S3	S1 vs S4	S2 vs S3	S2 vs S4	S3 vs S4
Isoprene	0.906760	0.001510**	0.984630	0.101600	0.999960	0.037160*	0.764200	0.800600	0.642960	0.044530*	1	0.024998*
C_5H_8O	1	2.40E-07***	2.51E-09***	1.16E-07***	1.51E-10***	0.995482	0.999993	0.990415	1	0.999245	0.999997	0.992841
Monoterpenes	1	4.51E-09***	0.360920	1.09E-09***	0.450940	<1E-307***	0.983690	0.999840	0.957480	0.999740	0.470430	0.784870
MACR+MVK	3.49E-06***	<1E-307***	4.10E-05***	0.108610	0.999650	0.026386*	0.001933**	0.729180	0.898190	0.000001***	0.125580	0.068677
Acetone	0.976040	6.72E-05***	0.457650	4.67E-07***	0.053418	0.111910	1.17E-04***	0.001936**	<1E-307***	<1E-307***	<1E-307***	8.48E-05
Acetaldehyde	1	0.996020	0.999690	0.997840	0.999880	1	0.058117	<1E-307***	<1E-307***	1.63E-09***	2.00E-09***	1
Butanone	0.930650	<1E-307***	5.89E-05***	<1E-307***	1.23E-07***	0.046285*	1	0.009892**	<1E-307***	0.007112**	<1E-307***	3.00E-04***
Pentanone	0.697740	0.033124*	0.002226**	0.805000	0.306480	0.994160	0.917220	0.004308**	1.27E-04***	2.03E-05***	2.42E-07***	0.989570
Methanethiol	0.662300	0.999660	0.981160	0.331830	0.993500	0.834940	3.28E-05***	0.012802*	0.002585**	0.830790	<1E-307***	<1E-307***
Dimethyl sulfide	0.974650	0.893950	0.907630	0.999980	0.999990	1	0.003774**	<1E-307***	0.001119**	0.022369*	0.99999	0.057034
$C_4H_{10}S$	1.49E-05***	<1E-307***	<1E-307***	0.096221	6.55E-09***	0.005804**	0.995120	7.73E-09***	<1E-307***	5.65E-07***	<1E-307***	0.793470
C_3H_8OS	5.10E-07***	<1E-307***	<1E-307***	<1E-307***	0.049861*	1.54E-06***	0.001696**	0.963450	1	0.062472	0.002618**	0.980300
Methanol	<1E-307***	<1E-307***	<1E-307***	7.85E-08***	1	7.87E-08***	<1E-307***	<1E-307***	0.993840	<1E-307***	<1E-307***	<1E-307***
Ethanol	0.999430	0.999550	0.989760	1	0.999990	0.999980	<1E-307***	<1E-307***	<1E-307***	0.998110	0.989210	0.801740
Methyl nitrite	7.73E-10***	<1E-307***	<1E-307***	0.20111	0.00233**	0.82827	0.999980	0.743880	3.68E-06***	0.522780	6.39E-07***	0.004617**

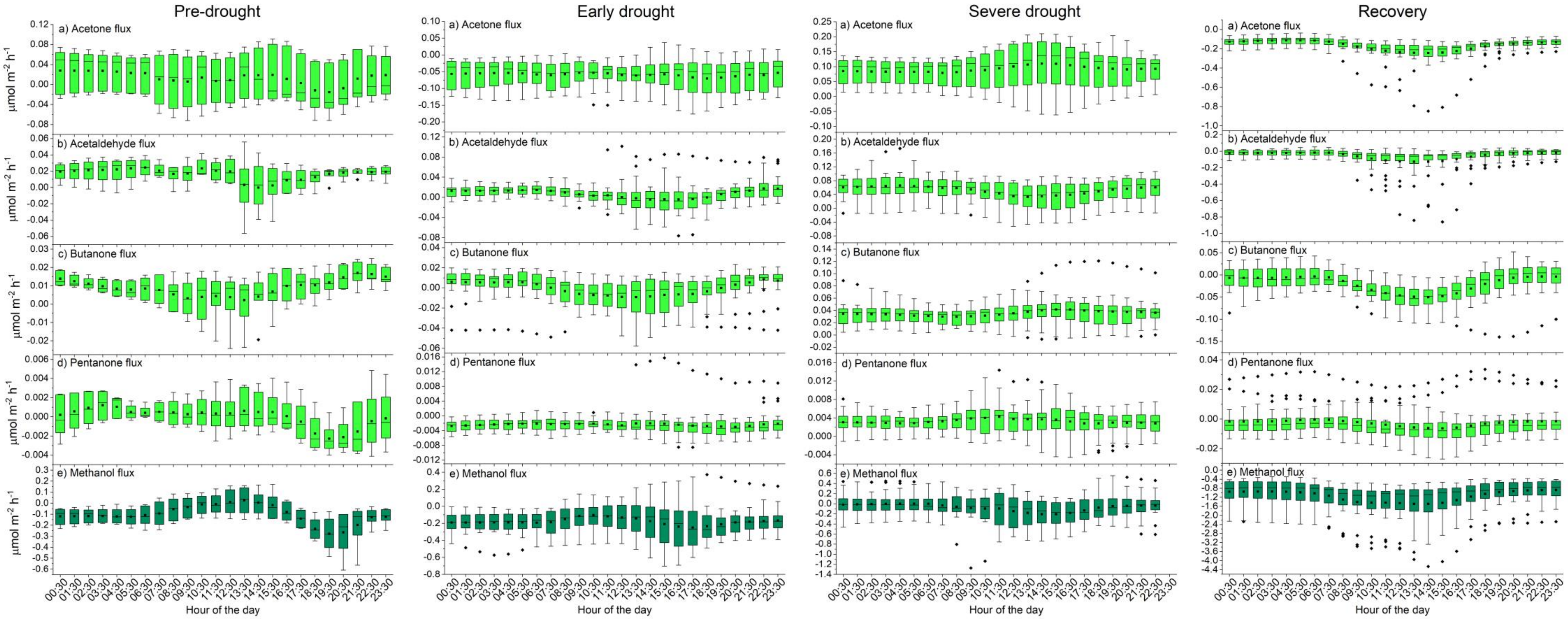

Figure S 5 Diel cycle of carbonyl compounds and methanol during the different period of the campaign. Diel cycle observed during pre-drought (n = 10 days), early drought (n = 26 days), severe drought (n = 27 days, the days of 13C-pyruvate experiments were excluded), and recovery (n = 21 days) for a) acetone, b) acetaldehyde, c) butanone, d) pentanone, and e) methanol soil fluxes. The boxes represent 25% to 75% of the dataset. The filled square and central lines indicate the mean and median values, respectively. The whiskers indicate the minimum and maximum data points at 1.5 times the interquartile range. Filled diamond indicate the outliers.

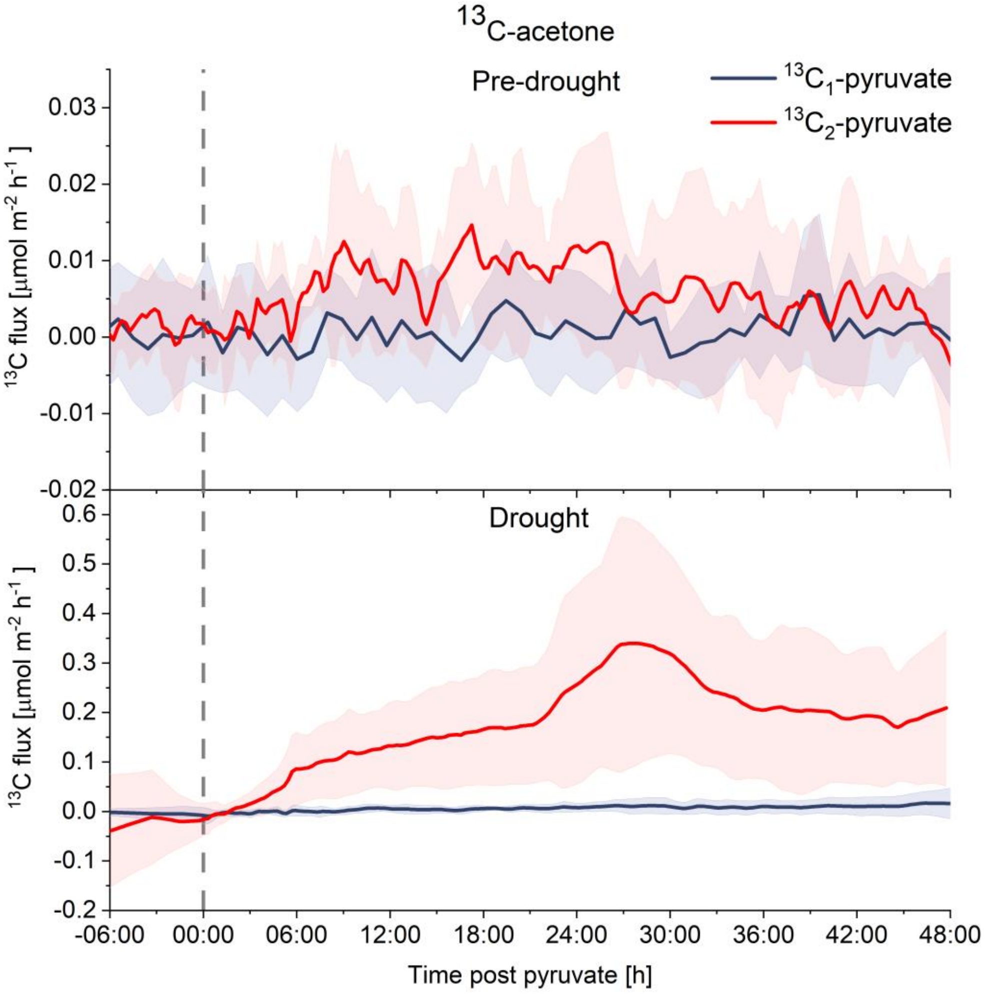

Figure S 6 Soil emissions of ^{13}C-acetone from 6 hours prior to 48 hours after ^{13}C-pyruvate soil injections. Soil emission fluxes of ^{13}C-acetone after the C_1-^{13}C-pyruvate (blue lines) and C_2-^{13}C-pyruvate (red lines) soil injections during pre-drought (upper panel) and drought (lower panel) period. Lines represent averaged fluxes over the 9 chambers (n = 9) subjected to ^{13}C-pyruvate injections. The shaded areas indicate the standard deviation.
