# Supplementary material for: Effects of drought and recovery on soil volatile organic compound fluxes in an experimental rainforest
Source: Nat Commun. 2023 Aug 21;14:5064. doi: 10.1038/s41467-023-40661-8 (PMC10442410; doi:10.1038/s41467-023-40661-8)
Supplement: Supplementary file 3 — Reporting summary [file 41467_2023_40661_MOESM3_ESM.pdf]

nature portfolio

Corresponding author(s): Giovanni Pugliese

Last updated by author(s): Jul 19, 2023

Reporting Summary

Nature Portfolio wishes to improve the reproducibility of the work that we publish. This form provides structure for consistency and transparency in reporting. For further information on Nature Portfolio policies, see our Editorial Policies and the Editorial Policy Checklist.

Statistics

For all statistical analyses, confirm that the following items are present in the figure legend, table legend, main text, or Methods section.

n/a	Confirmed	
☐	☒	The exact sample size (n) for each experimental group/condition, given as a discrete number and unit of measurement
☐	☒	A statement on whether measurements were taken from distinct samples or whether the same sample was measured repeatedly
☐	☒	The statistical test(s) used AND whether they are one- or two-sided<br>*Only common tests should be described solely by name; describe more complex techniques in the Methods section.*
☐	☒	A description of all covariates tested
☐	☒	A description of any assumptions or corrections, such as tests of normality and adjustment for multiple comparisons
☐	☒	A full description of the statistical parameters including central tendency (e.g. means) or other basic estimates (e.g. regression coefficient) AND variation (e.g. standard deviation) or associated estimates of uncertainty (e.g. confidence intervals)
☐	☒	For null hypothesis testing, the test statistic (e.g. F, t, r) with confidence intervals, effect sizes, degrees of freedom and P value noted<br>*Give P values as exact values whenever suitable.*
☒	☐	For Bayesian analysis, information on the choice of priors and Markov chain Monte Carlo settings
☒	☐	For hierarchical and complex designs, identification of the appropriate level for tests and full reporting of outcomes
☒	☐	Estimates of effect sizes (e.g. Cohen's d, Pearson's r), indicating how they were calculated

Our web collection on statistics for biologists contains articles on many of the points above.

Software and code

Policy information about availability of computer code

Data collection	ToFDaq Viewer version 1.2.93.
Data analysis	PTRwid (verion V003) for PTR-ToF data analysis, OriginPro (version 2021b) for PLSR analysis and Tukey mean comparison test, package chngpt R (version 4.1.1) for soil moisture threshold model.

For manuscripts utilizing custom algorithms or software that are central to the research but not yet described in published literature, software must be made available to editors and reviewers. We strongly encourage code deposition in a community repository (e.g. GitHub). See the Nature Portfolio guidelines for submitting code & software for further information.

Data

Policy information about availability of data

All manuscripts must include a data availability statement. This statement should provide the following information, where applicable:

- Accession codes, unique identifiers, or web links for publicly available datasets
- A description of any restrictions on data availability
- For clinical datasets or third party data, please ensure that the statement adheres to our policy

All data that support the findings of this study are publicly available in FigShare (https://doi.org/10.6084/m9.figshare.22770782)

Research involving human participants, their data, or biological material

Policy information about studies with human participants or human data. See also policy information about sex, gender (identity/presentation), and sexual orientation and race, ethnicity and racism.

Reporting on sex and gender	N/A
Reporting on race, ethnicity, or other socially relevant groupings	N/A
Population characteristics	N/A
Recruitment	N/A
Ethics oversight	N/A

Note that full information on the approval of the study protocol must also be provided in the manuscript.

Field-specific reporting

Please select the one below that is the best fit for your research. If you are not sure, read the appropriate sections before making your selection.

☐ Life sciences ☐ Behavioural & social sciences ☒ Ecological, evolutionary & environmental sciences

For a reference copy of the document with all sections, see nature.com/documents/nr-reporting-summary-flat.pdf

Ecological, evolutionary & environmental sciences study design

All studies must disclose on these points even when the disclosure is negative.

Study description	In this study we assess the impact of prolonged drought followed by rain and recovery on soil VOC fluxes from the tropical rainforest mesocosm of the Biosphere 2 (B2 TRF). From September 2019 to January 2020 the experimental rainforest was subjected to 65 days of controlled drought followed by by rain and recovery period. The soil VOC fluxes were measured continuously over the whole campaign from 12 automated soil chambers placed in 4 different location (3 for each location) in the experimental rainforest. Soil respiration, soil temperature, soil moisture, and soil matric potential were also measured simultaneously to the soil VOC fluxes.
Research sample	The VOC fluxes were measured from 12 soil chambers placed in 4 different locations representing the soil heterogeneity within the experimental rainforest.
Sampling strategy	Gas samples for soil VOC fluxes measurement were sampled continuously by means of the inlet of a proton transfer reaction mass spectrometer (PTR-TOF-MS) directly connected to the soil chambers system. No sample size calculation was performed as 3 replicate chambers for each of the 4 sites (total 12 chambers) were accepted to be sufficient to perform statistical analysis.
Data collection	The VOC data were automatically recorded by the acquisition software of the PTR-TOF-MS, ToFDaq Version 1.2.93. The PTR-ToF instrument was operated by Thomas Klüpfel and Eva Y. Pfannerstill.
Timing and spatial scale	VOC data were acquired from 01/09/2019 to 03/01/2020. VOC concentrations were measured every 10s to capture fast VOC concentration changes. Soil VOC fluxes measurement from one chamber lasted 10 minutes: 2.5 minutes chamber pre-purge to flush the system lines, 6.5 minutes chamber closure to ensure VOC flux measurement could be performed, 1 minute post purge to flush the system line. All 12 chambers were measured consecutively resulting in a temporal resolution of 2 hours. The 12 chambers were placed in group of 3 in 4 different sites. The sites were on average 25 m away from each other (minimum distance about 10 m and maximum distance about 36 m) and the chambers within each site were placed on average 2.5 m apart (minimum distance about 1.5 m and maximum distance about 4.5 m).
Data exclusions	VOC data collected from 1st to 11th September were excluded from the manuscript as during this period we performed several tests for the optimization of the setup. On 11th September we started with the 13C-pyruvate labeling experiments.
Reproducibility	The study was a long term (97 days) experiment during which 65 days of drought were induced in the experimental rainforest. As it was a large scale field experiment, the exact conditions cannot be reproduced.
Randomization	It is not applicable to our study as all 12 soil chambers were subjected to the same treatment (drought) at the same time, and each chamber was measured with a temporal resolution of 2 hours over the whole campaign (97 days).
Blinding	It is not applicable to our study as it was a field experiment and all the investigators knew about the drought treatment.

Did the study involve field work? ☒ Yes ☐ No

Field work, collection and transport

Field conditions	Within the tropical rainforest mesocosm of the Biosphere 2 the temperature range was between 21 and 37 °C (day) and 20 and 27 °C (night). The precipitations were controlled.
Location	Biosphere 2, University of Arizona, Oracle, AZ, USA 32°34′43.6″N, 110°51′02.14″W, 1164 m above mean sea level
Access & import/export	The Biosphere 2 is an accessible experimental facility where no export/import control are require. Moreover we did not export any samples and the work was conducted in collaboration with researcher of the Biosphere 2.
Disturbance	No relevant disturbance

Reporting for specific materials, systems and methods

We require information from authors about some types of materials, experimental systems and methods used in many studies. Here, indicate whether each material, system or method listed is relevant to your study. If you are not sure if a list item applies to your research, read the appropriate section before selecting a response.

Materials & experimental systems

n/a	Involved in the study	
☒	☐	Antibodies
☒	☐	Eukaryotic cell lines
☒	☐	Palaeontology and archaeology
☒	☐	Animals and other organisms
☒	☐	Clinical data
☒	☐	Dual use research of concern
☒	☐	Plants

Methods

n/a	Involved in the study	
☒	☐	ChIP-seq
☒	☐	Flow cytometry
☒	☐	MRI-based neuroimaging
